# Supplementary material for: Molecular Characterization of Outer Capsid Proteins VP5 and VP7 of Grass Carp Reovirus
Source: Viruses. 2022 May 12;14(5):1032. doi: 10.3390/v14051032 (PMC9148132; doi:10.3390/v14051032)
Supplement: Supplementary file 1 [file viruses-14-01032-s001.zip › viruses-1721129-supplementary.pdf]

Supplementary materials

Table S1. Identification of peptide fragments generated from tryptic digestion of GCRV-VP5

No.	Start–End	Peptide Sequence	Length	*m/z* (Observed)	*ppm*
1	43–67	PTGKLWRPVGTSVATIDSLAIVSDR	25	880.4876	–1
2	43–79	PTGKLWRPVGTSVATIDSLAIVSDRFGQYSFVNEGMR	37	1,014.5255	0
3	47–67	LWRPVGTSVATIDSLAIVSDR	21	1,128.6216	–1
4	47–79	LWRPVGTSVATIDSLAIVSDRFGQYSFVNEGMR	33	922.7188	–1
5	47–84	LWRPVGTSVATIDSLAIVSDRFGQYSFVNEGMRETFSK	38	1,070.7909	–1
6	49–67	RPVGTSVATIDSLAIVSDR	19	979.0374	–2
7	50–67	PVGTSVATIDSLAIVSDR	18	900.9891	0
8	50– 79	PVGTSVATIDSLAIVSDRFGQYSFVNEGMR	30	1,072.8648	–5
9	68–79	FGQYSFVNEGMR	12	717.8276	0
10	68–84	FGQYSFVNEGMRETFSK	17	1,021.9659	–1
11	80–100	ETFSKALFDINMWQPLFQATK	21	844.4253	–1
12	85–100	ALFDINMWQPLFQATK	16	646.9977	0
13	123–147	AGDALDNPVTNGVFISTVQIMNLQR	25	891.7945	1
14	126–147	ALDNPVTNGVFISTVQIMNLQR	22	810.7610	–5
15	153–161	MRDVALWQK	9	573.8072	1
16	155–161	DVALWQK	7	430.2374	0
17	162–184	HLDTAMTMLTPDISAGSASCNWK	23	844.0332	0
18	185–191	SLLAFAK	7	375.2319	0
19	192–214	DILPLDNLCLTYPNEFYNVAIHR	23	938.4453	–5
20	201–214	LTYPNEFYNVAIHR	14	579.6302	0
21	215–226	YPALKPGNPDTK	12	434.2342	–1
22	215–243	YPALKPGNPDTKLPDAQAHPLGEVAGAFN	29	996.8448	1
23	244–269	AATSEVGSLVGSSSTLSQAISTMAGK	26	1,220.6155	0
24	268–292	GKDLDLIEADTPLPVSVFTPSLAPR	25	884.4814	0
25	270–292	DLDLIEADTPLPVSVFTPSLAPR	23	849.4308	0
26	293–318	SYRPAFIKPEDAKWIAEFNNSSLIRK	26	616.9316	–1
27	319–337	TLTYSGATYTVQLGPGPTR	19	992.0123	0
28	404–412	AINLAINVR	9	492.3017	1
29	417–428	FNMLHLQATFER	12	513.6077	0
30	444–472	LIPSPTSSSNFSNPTLMDGLLTVTPVLLR	29	1,029.5585	5
31	473–516	ETTYKGEVVDAIVPATVMANQTSEEVASALANDAIVLVSNHLNK	44	1,153.8408	–1
32	517–542	LANVVGDAIPVASRTDDSATSAIVSR	26	1,293.1757	–3
33	543–567	LAVQHKLSQVGQASPTPPDYPLLWR	25	701.1326	0
34	543–568	LAVQHKLSQVGQASPTPPDYPLLWRR	26	740.1588	1
35	571–609	RAASMFVSNPSLALQVGIPVLTQSGMLSALTSGVGTALR	39	1,301.0393	0
36	610–625	TGSLGKGVTDASEKLR	16	540.2968	0
37	626–648	ARQSLTVAKQAFFDQIGSLWPGK	23	850.1263	–1
38	635–648	QAFFDQIGSLWPGK	14	797.4063	0

m/z: mass charge ratio

Table S2. Identification of peptide fragments generated from tryptic digestion of GCRV-VP7

**No.**	**Start–End**	**Peptide Sequence**	**Length**	***m*/*z*** **(Observed)**	***ppm***
1	28–45	TRTETTNFDHAEYVTCGR	18	634.2912	5
2	64–80	TIQDSHACSRQPNEAIR	17	1,008.005	–1
3	81–88	SLVEVSDK	8	438.7404	2
4	81–97	SLVEVSDKAQTALVGSR	17	587.3347	4
5	98–107	TVDYHELDVK	10	406.8809	–2
6	108–123	AGFVAPTADETIAPSK	16	787.9172	3
7	124–131	DIVELPFR	8	494.7855	4
8	132–145	TCDLDDSSATACVR	14	785.8324	1
9	146–166	NHCQAGHDGVIHLPILSGDFK	21	595.5674	1
10	167–184	LPNEHPTKPLDDTHPHDK	18	547.5541	0
11	192–214	TGLLLVHDTHAHATAVVATAATR	23	582.3337	5
12	215–234	AILMHDLLTSANADDGHQAR	20	538.0168	1

m/*z*: mass charge ratio
